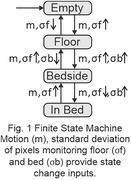# Non‐imaging Passive Infrared Matrix Bed Occupancy Sensor for Persons with Dementia

**DOI:** 10.1002/alz70858_098160

**Published:** 2025-12-24

**Authors:** Aragondram Kiran Kumar, Raheem Qaiser, Ali Gulsatar, Xuanda Chen, Jonathon David White

**Affiliations:** ^1^ Yuan Ze University, Taoyuan, Taoyuan, Taiwan; ^2^ Yuan Ze University, Zhong‐Li, Taoyuan, Taiwan; ^3^ Thomas A Blakelock High School, Oakville, ON, Canada; ^4^ McMaster University, Hamilton, ON, Canada

## Abstract

**Background:**

65% of persons with dementia (PWD) experience disturbed sleep, often exiting their beds up to 14 times per night, impacting caregivers' well‐being. The unpredictability is a leading cause of institutionalization in Taiwan. Reliable sleep monitoring can help caregivers rest better, reducing their stress. Current sensors include mechanical pressure sensors and cameras. Pressure sensors offer privacy but require mattress contact. Since PWDs sleep may sleep in unusual postures, comprehensive coverage while necessary, is costly and may be uncomfortable. Cameras, while contact‐free, raise privacy issues and don’t work well with heavy blankets. We developed a bed occupancy sensor using a passive infrared matrix sensor that maintains privacy, costs less than $100/unit, and works with blankets, quilts, and unusual sleeping positions of PWD.

**Method:**

An Infrared matrix and a motion sensor on the ceiling monitor the room. Standard deviations of bed, floor and motion data is processed by a microcontroller, which determines if the PWD is in bed (sleeping soundly or fitfully), sitting on the side, beside the bed, or has left the room.

**Results:**

The system was tested in the lab, detecting bed entries and exits under various conditions with < 5s response time, using a commercial bed pressure sensor as a benchmark. It was also deployed for 136 days in a real‐life setting in Taiwan, where temperatures ranged from 8°C to 35°C.

**Conclusion:**

An ambient temperature insensitive, low‐cost cost, privacy‐preserving, non‐imaging remote bed occupancy sensor, and algorithm has been developed with < 5s response time.